# Disease activity indexes might not capture the same disease aspects in males and females with ankylosing spondylitis: A real-world nationwide analysis

**DOI:** 10.3389/fmed.2022.1078325

**Published:** 2022-12-21

**Authors:** Cristina Fernández-Carballido, Vega Jovaní, Emma Beltrán Catalán, Manuel José Moreno-Ramos, Jesús Sanz Sanz, Adela Gallego, M. Luz García Vivar, José Manuel Rodríguez-Heredia, Cristina Sanabra, Carlos Sastré

**Affiliations:** ^1^Department of Rheumatology, Hospital Universitario San Juan de Alicante, Alicante, Spain; ^2^Department of Rheumatology, Hospital General Universitario Dr. Balmis, Alicante Institute of Health and Biomedical Research (ISABIAL), Alicante, Spain; ^3^Department of Rheumatology, Hospital del Mar, Barcelona, Spain; ^4^Department of Rheumatology, Hospital Clínico Universitario Virgen de la Arrixaca, Murcia, Spain; ^5^Department of Rheumatology, Hospital Universitario Puerta de Hierro, Madrid, Spain; ^6^Department of Rheumatology, Complejo Hospitalario Universitario de Badajoz, Badajoz, Spain; ^7^Department of Rheumatology, Hospital Universitario Basurto, Bilbao, Spain; ^8^Department of Rheumatology, Hospital Universitario de Getafe, Madrid, Spain; ^9^Novartis Farmacéutica, Barcelona, Spain

**Keywords:** gender, disease activity, ankylosing spondylitis (AS), health status, BASDAI, ASDAS-CRP, ASAS-HI

## Abstract

**Background:**

To evaluate gender differences in disease activity and health status (HS) in patients with radiographic axial spondyloarthritis (r-axSpA)/ankylosing spondylitis (AS).

**Methods:**

Ancillary analysis of the MIDAS study, an observational, non-interventional, cross-sectional and retrospective multicenter nationwide study to assess disease activity and its relationship with HS in clinical practice. Adult patients with AS diagnosis, fulfilling ASAS and modified New York criteria, treated for ≥3 months upon study inclusion according to clinical practice were included. The primary outcome was “disease control” assessed by the percentage of patients in remission and low disease activity (BASDAI and ASDAS-CRP scores). HS was evaluated using the ASAS health index (ASAS-HI). Patients' responses and characteristics were analyzed by gender.

**Results:**

We analyzed 313 patients with AS, 237 (75.7%) males and 76 (24.3%) females. A total of 202 (64.5%) patients had adequate disease control (BASDAI < 4); 69.2% of males [mean (SD) BASDAI 2.9 (2.1)] and 50.0% of females [mean (SD) BASDAI 3.8 (2.4); *p* = 0.01]. According to ASDAS-CRP, 57.5% of patients were adequately controlled (ASDAS-ID +ASDAS-LDA); 138 (58.2%) males and 42 (55.3%) females. The mean (SD) ASDAS-CRP was 1.9 (1.1); being 1.9 (1.0) in males and 2.0 (1.1) in females. Overall, the impact of AS on HS was low to moderate [mean (SD) ASAS-HI 5.8 (4.4)]; being 5.5 (4.4) for males and 6.8 (4.2) for females (*p* = 0.02).

**Conclusion:**

This study showed a higher proportion of females with AS and active disease using the BASDAI definition. When using the ASDAS-CRP definition these differences by gender were less pronounced. The impact of disease activity on HS appears to be higher in females than males.

## 1. Introduction

The term spondyloarthritis (SpA) encompasses several inflammatory diseases that share epidemiological, pathogenic, genetic, clinical, radiographic, and therapeutic response features. Usually, SpA are divided into axial SpA (axSpA) and peripheral SpA (pSpA) forms, depending on the predominant clinical manifestations. Within axSpA, the Ankylosing Spondylitis Assessment Society (ASAS) classification criteria defines two forms: ankylosing spondylitis (AS) or radiographic axSpA (r-axSpA) and non-radiographic axSpA (nr-axSpA) (1, 2), classified by the presence/absence of radiographic sacroiliitis, according to the modified New York criteria. Patients suffering from axSpA should be treated effectively because the disease can lead to irreversible damage of the spine and joints, resulting in chronic pain, disability, and a negative impact on the patient's quality of life (3).

The female prevalence is still low in studies of patients with axSpA and even lower in those with AS, and analyses stratified by gender are often not performed nor disclosed (4). Available studies on gender differences in patients with axSpA point out that females have different disease manifestations, and that disease activity (measured through the Bath Ankylosing Spondylitis Disease Activity Index, BASDAI) and quality of life (by using the Ankylosing Spondylitis Quality of Life index, ASQoL) are significantly worse in females (4, 5). Also, some studies suggest that response to treatment varies by gender, with female patients having lower response rates and lower survival and adherence to treatments such as tumor necrosis factor inhibitors (TNFi) (4–6) or secukinumab (7). Several studies on gender differences in patients with axSpA have revealed biological mechanisms which could hypothetically contribute to the observed differences in disease manifestations and treatment response (4). For example, the serum levels of interleukin (IL) 17A (IL-17A) and TNF are significantly higher, and T helper-17 cells are also increased in male patients with AS but not in females (8). Also, amongst AS patients with syndesmophytes, males had significantly higher IL-18 levels, whereas females showed significantly elevated IL-6 (9). For this reason, a greater understanding of the differences between female and male pathogenesis and clinical manifestations is needed. The present study consists of an ancillary analysis of the MIDAS study to evaluate gender differences in disease activity and health status (HS).

## 2. Materials and methods

### 2.1. Study design

MIDAS is an observational, non-interventional, descriptive, cross-sectional, retrospective, and multicenter study. The study was conducted in 36 centers with outpatient rheumatology clinics in Spanish public hospitals between December 10^*th*^, 2018, and August 14^*th*^, 2019. Two different cohorts were included: 313 patients with AS and 313 patients with psoriatic arthritis. The inclusion and exclusion criteria, study design, study procedures and main results have already been published (10). The main objective of the MIDAS study was to evaluate the percentage of patients with controlled disease activity. Here we present the results of an ancillary analysis to evaluate gender differences in disease activity and HS in the patients with AS.

The Spanish version of the BASDAI was used to assess disease activity (range 0–10). In the MIDAS study, a BASDAI score < 4 was considered low disease activity and a BASDAI score ≤ 2 as disease remission (11). Disease control was defined as reaching at least a BASDAI of low disease activity (10). Disease activity was also measured through the Ankylosing Spondylitis Disease Activity Score index using C-reactive protein (ASDAS-CRP), and the percentage of patients with an ASDAS-CRP score < 2.1 (low disease activity) and < 1.3 (inactive disease) was calculated. Disease control was defined as reaching at least an ASDAS-CRP of low disease activity. Additionally, HS was evaluated using the Spanish version of the ASAS health index (ASAS-HI) and the patients' acceptable symptom state (PASS) (12) was used to evaluate the level of symptoms at which patients consider themselves well.

The study was conducted according to Good Clinical Practice (International Conference of Harmonization) guidelines, the Declaration of Helsinki, and local regulations, including privacy laws, at the time of the initiation of the study. Thus, the study was performed according to the guidelines on observational post-authorization studies for medicinal products for human use (Order SAS/3470/2009) of the Spanish Agency of Medicines and Medical Devices (AEMPS). The Ethical and Clinical Research Committee of the 12 de Octubre Hospital approved the study protocol, informed consent forms, and patient information (approval number 18/437) (10).

### 2.2. Statistical analysis

Continuous variables were described by mean and standard deviation (SD) and categorical variables by number and percentages. Descriptive analysis was based on evaluable data per parameter, excluding patients with missing values. Patients' characteristics and disease activity were compared between males and females. For continuous variables, two-sample *t*-tests (for normally distributed variables) or the Wilcoxon test (for non-normally distributed variables) were used. For categorical variables Chi-square tests were employed. The level of significance was set as a two-tailed p < 0.05. Data were analyzed with Statistical Analysis System Enterprise Guide 7.15 (10).

## 3. Results

### 3.1. Baseline characteristics

A total of 336 subjects with AS were recruited in the MIDAS Study. Of them, 313 (93.2%) were eligible. Table 1 depicts the patients' baseline characteristics overall and divided by gender. Briefly, mean (SD) age was 50.4 (12.0) years, and 75.7% of them were male. No gender differences were identified regarding diagnosis delay (evaluated as the years since the onset of symptoms to diagnosis of AS) or the age at the time of the study.

**Table 1:** Baseline demographic and clinical characteristics of the patients with AS analyzed in the MIDAS study.

	**Total**	**Male**	**Female**	***p*-value**
	**(*n* = 313)**	**(*n* = 237)**	**(*n* = 76)**	
Age (years), mean (SD)	50.4 (12.0)	50.1 (12.2)	51.2 (11.5)	0.32
Years since AS diagnosis, mean (SD)	15.5 (11.6)	16.8 (12.2)	11.4 (8.5)	**0.01**
Years since the AS symptoms' onset to the study visit, mean (SD)	20.5 (12.7)	22.2 (13.0)	15.2 (9.9)	**< 0.01**
Years since the AS symptoms' onset to diagnosis, mean (SD)	5.0 (7.2)	5.4 (7.7)	3.9 (5.6)	0.39
**BMI (kg/m** ^2^ **), mean (SD)**	27.0 (4.9)	27.5 (4.6)	25.5 (5.6)	**0.01**
Obesity (BMI>30), n (%)	67 (23.0)	53 (23.7)	14 (20.9)	
**Smoking habit**				**0.03**
Current smoker, n (%)	75 (24.0)	61 (25.7)	14 (18.4)	
Ex-smoker (>6 months), n (%)	81 (25.9)	68 (28.7)	13 (17.1)	
Non-smoker, n (%)	137 (43.8)	96 (40.5)	41 (53.9)	
Family history of AS/PsA, n (%)	66 (21.1)	48 (20.3)	18 (23.7)	0.71
Presence of HLA-B27, n (%)	245 (78.5)	187 (79.2)	58 (76.3)	0.77
Patients previously treated with bDMARDs, n (%)	99 (31.6)	77 (32.5)	22 (28.9)	0.63
**Treatments at the moment of the study visit, n (%)**				
Patients treated with a combination of bDMARDs and nbDMARDs	136 (43.5)	102 (43.0)	34 (44.7)	-
Patients treated with bDMARDs	229 (73.2)	179 (75.5)	50 (65.8)	0.10
**TNFi**	201 (87.8)	156 (87.2)	45 (90)	-
Adalimumab	73 (36.3)	57 (24.1)	16 (21.1)	0.59
Etanercept	45 (22.3)	38 (16.0)	7 (9.2)	0.14
Infliximab	29 (14.4)	22 (9.3)	7 (9.2)	0.99
Golimumab	37 (18.4)	30 (12.7)	7 (9.2)	0.42
Certolizumab pegol	17 (8.46)	9 (3.8)	8 (10.5)	**0.02**
IL-23i: Ustekinumab	2 (0.9)	1 (0.4)	1 (1.3)	0.40
IL-17i: Secukinumab	26 (11.3)	22 (9.3)	4 (5.3)	0.27
**Other treatments** ^*^	220 (70.3)	160 (67.5)	60 (78.9)	0.06
NSAIDs	168 (76.4)	120 (50.6)	48 (63.2)	0.06
nbDMARDs	70 (31.8)	50 (21.1)	20 (26.3)	0.34
**Active disease, n (%)** ^**^				
BASDAI ≥4	111 (35.5)	73 (30.8)	38 (50.0)	**0.01**
ASDAS-CRP ≥2.1	133 (42.4)	99 (41.8)	34 (44.7)	0.65
CRP levels (mg/l), mean (SD)	5.1 (8.2)	5.7 (9.0)	3.3 (4.3)	0.07
Peripheral disease, n (%)^***^	62 (19.8)	44 (18.6)	18 (23.7)	**0.05**

AS, ankylosing spondylitis; ASDAS, Ankylosing Spondylitis Disease Activity Score; BASDAI, Bath Ankylosing Spondylitis Disease Activity Index; bDMARD, biologic disease modifying anti-rheumatic drug; BMI, body mass index; CRP, C-reactive protein; HLA-B27, human leukocyte antigen B27; IL-17i, interleukin 17 inhibitors; IL-23i, interleukin 23 inhibitors; nbDMARD, non-biologic disease modifying anti-rheumatic drug; NSAID, non-steroidal anti-inflammatory drug; PsA, psoriatic arthritis; SD, standard deviation; TNFi, tumor necrosis factor inhibitors.

Bold values are the significative ones according to our study statistics definitions.

^*^Alone or in combination. ^**^Refers to the percentage of patients with active disease according to BASDAI≥4 and ASDAS-CRP≥2.1. ^***^Peripheral disease was defined as presence of peripheral arthritis as disclosed by the investigators of the participating centers.

### 3.2. Disease activity control

In the whole sample (Table 2), the mean (SD) BASDAI score was 3.1 (2.2) and 64.5% of the patients showed adequate disease control (BASDAI < 4), while 38% were in remission (BASDAI ≤ 2). The mean (SD) ASDAS-CRP score was 1.9 (1.1). According to ASDAS-CRP cut-off points, 29.4% of the patients had inactive disease (ASDAS-CRP < 1.3), 28.1% low disease activity (1.3 ≤ ASDAS-CRP < 2.1), 33.5% high disease activity (2.1 ≤ ASDAS-CRP < 3.5), and 8.9% very high disease activity (ASDAS-CRP ≥ 3.5). Overall, the impact on HS (ASAS-HI) was low to moderate, with a mean (SD) ASAS-HI of 5.8 (4.4) and 86.3% of the patients declared an acceptable symptom (PASS).

**Table 2:** Disease control, health status and patient's acceptable symptom state of the patients with AS in the MIDAS study, by gender.

	**Total**	**Male**	**Female**	***p*-value**
	**(*n =* 313)**	**(*n =* 237)**	**(*n =* 76)**	
BASDAI, mean (SD)	3.1 (2.2)	2.9 (2.1)	3.8 (2.4)	**0.01**
BASDAI < 4, n (%)	202 (64.5)	164 (69.2)	38 (50.0)	**0.01**
BASDAI ≤ 2, n (%)	119 (38.0)	97 (40.9)	22 (28.9)	0.06
BASDAI in patients with peripheral involvement, mean (SD)	4.2 (2.4)	3.7 (2.4)	5.2 (2.1)	**0.02**
ASDAS-CRP, mean (SD)	1.9 (1.1)	1.9 (1.0)	2.0 (1.1)	0.60
**ASDAS-CRP disease activity categories**				0.92
ASDAS-CRP < 1.3, n (%)	92 (29.4)	69 (29.1)	23 (30.3)	
ASDAS-CRP ≥1.3 < 2.1, n (%)	88 (28.1)	69 (29.1)	19 (25.0)	
ASDAS-CRP ≥2.1 < 3.5, n (%)	105 (33.5)	78 (32.9)	27 (35.5)	
ASDAS-CRP ≥3.5, n (%)	28 (8.9)	21 (8.9)	7 (9.2)	
ASDAS-CRP in patients with peripheral involvement, mean (SD)	2.4 (1.1)	2.4 (1.2)	2.5 (0.9)	0.50
ASAS-HI, mean (SD)	5.8 (4.4)	5.5 (4.4)	6.8 (4.2)	**0.02**
PASS, n (%)	270 (86.3)	208 (87.8)	62 (81.6)	0.17

AS, ankylosing spondylitis; ASAS-HI, Assessment of Spondyloarthritis International Society - Health index; ASDAS, Ankylosing Spondylitis Disease Activity Score; BASDAI, Bath Ankylosing Spondylitis Disease Activity Index; PASS, patient acceptable symptom state; SD, standard deviation.

Bold values are the significative ones according to our study statistics definitions.

**Figure 1:**
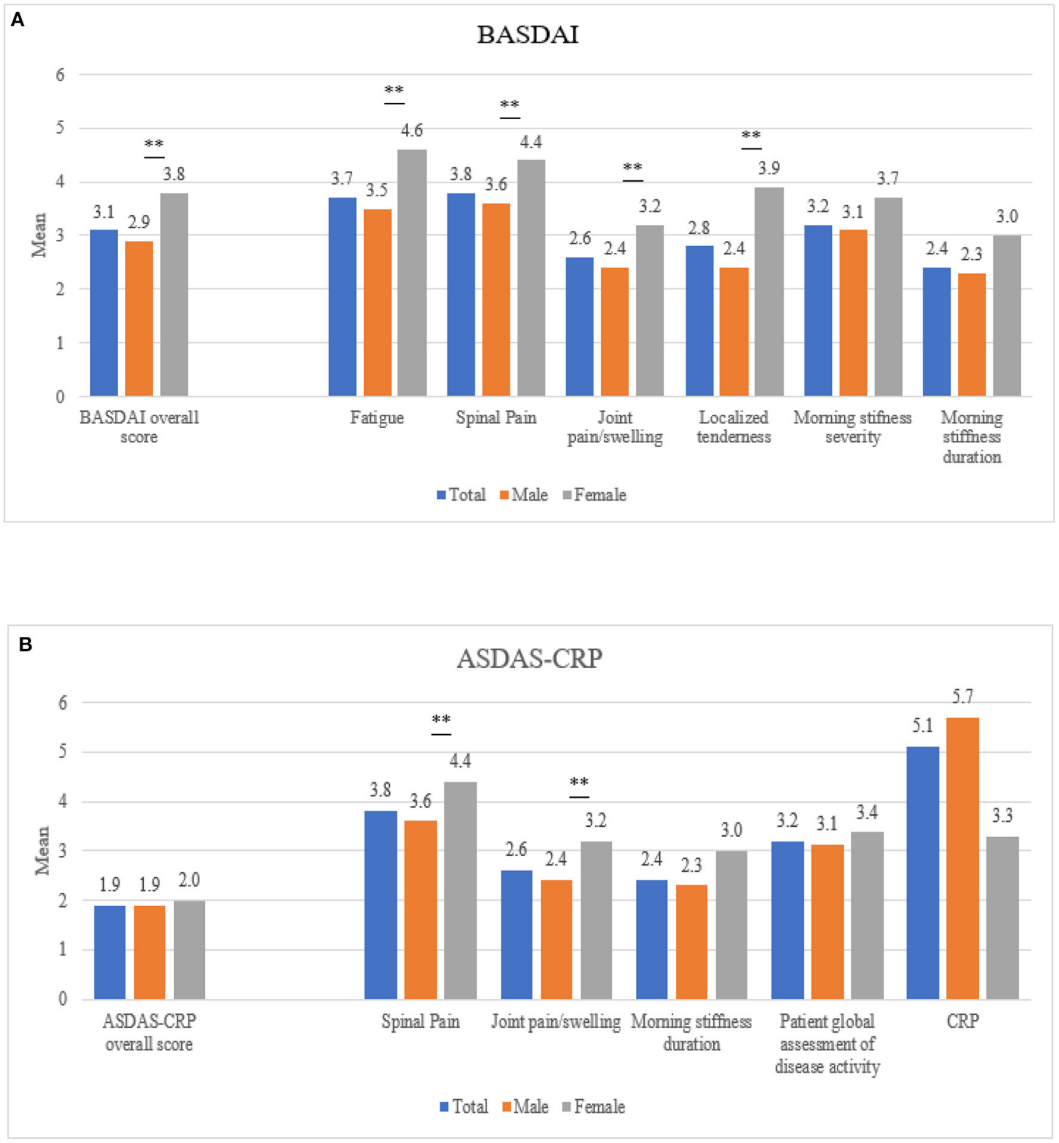
Mean disease activity scores by gender, measured by **(A)** BASDAI and **(B)** ASDAS-CRP. BASDAI, Bath Ankylosing Spondylitis Disease Activity Index; ASDAS-CRP, Ankylosing Spondylitis Disease Activity Score with CRP. The left values on x-axis denotes the mean index score and values on the right of the x-axis display values for each individual component of the indices. **p < 0.05.

According to BASDAI, disease activity was controlled in 69.2% of the male patients and in 50.0% of the female patients (*p* = 0.01). Consequently, mean (SD) BASDAI was lower for males [2.9 (2.1)] than for females [3.8 (2.4); *p* = 0.01] (Table 2; Figure 1A). Regarding the different BASDAI score items, females had significatively higher scores for the items B1 (fatigue), B2 (spinal pain), B3 (joint pain/swelling) and B4 (localized tenderness), without differences regarding morning stiffness (B5 and B6) (Figure 1A).

According to ASDAS-CRP, 58.2% of males and 55.3% of females showed low disease activity (Table 2; Figure 1B).

The mean (SD) impact on HS, evaluated through ASAS-HI, was 5.5 (4.4) for males and 6.8 (4.2) for females (*p* = 0.02) and a PASS was reported in 87.8% of males and 81.6% of females (Table 2).

When analyzing disease control according to the presence/absence of peripheral disease, the percentage of patients with disease control (BASDAI < 4) was lower for those with peripheral involvement (41.9%) vs. the subgroup without peripheral manifestations (70.1%). Indeed, among individuals with peripheral involvement the mean BASDAI score was significantly higher in females [5.2 (2.1)] than in males [3.7 (2.4); *p* = 0.02].

## 4. Discussion

The MIDAS study was designed to assess the disease activity state of patients with AS treated in routine clinical practice. This ancillary analysis evaluated gender differences in disease activity and HS in these patients. Overall, our results showed that disease activity in females was less properly controlled, by means of a higher proportion of females with active disease when using the BASDAI definition and a higher impact on HS measured through ASAS-HI. In the European Map of Axial Spondyloarthritis (EMAS) study across 13 countries, females reported a higher degree of disease activity in all BASDAI aspects (13). In line with these results, our analysis showed a higher proportion of females with active disease using the BASDAI definition.

However, fewer differences in disease activity were detected when using the ASDAS-CRP definition. In this regard, a recent systematic review and metanalysis showed that BASDAI was higher in females, but no differences in the ASDAS were detected, suggesting that the two most widely used indices of disease activity in axSpA discriminate differently according to gender (14), as we disclosed in our study. The authors proposed that this is most likely because of their differences in their components and the relative weight of them in the final score, which would result in different BASDAI and ASDAS scores depending on the presence/absence of different disease manifestations (14). Females in our study had more peripheral manifestations and significatively higher scores for fatigue, spinal pain, joint pain/swelling and localized tenderness than males. In this sense, fatigue is known to be more frequent and intense among females (4) and might be one of the explanations for higher BASDAI scores in female patients. Regarding peripheral manifestations, the question about joint pain/swelling has a lower weight in the ASDAS score than in the BASDAI index and the presence of enthesitis, more frequent and severe in females, is not included in ASDAS, but it is in BASDAI, as the fourth question of BASDAI is considered as a proxy for enthesitis (15). All these clinical differences could at least partially explain why ASDAS may not be sensitive enough to detect gender differences in disease activity (14, 16). We acknowledge that ASDAS is the preferred and most recommended index to evaluate disease activity nowadays, for different reasons (17). However, when the above-mentioned manifestations are predominant, the ASDAS could be underestimating disease activity. For this reason, we believe that is important to consider the disclosed differences in these indexes when evaluating the patients with AS, especially females.

Furthermore, concomitant conditions can also impact disease activity scores. For instance, both pain and enthesitis can be seen in patients with fibromyalgia, which could lead to inaccurately higher overall BASDAI scores in patients with axSpA and concomitant fibromyalgia, more frequently found in females with axSpA than in males (16, 18).

Likewise, while fatigue is a relevant manifestation in patients with SpA and is usually higher in females, it may also be increased because of other conditions, such as mental health disorders and sleep disturbances (19). Since females with SpA report greater psychological distress than males, measuring disease activity with BASDAI could lead to greater differences between males and females. In fact, higher BASDAI scores have been associated with depression and suggest the presence of fibromyalgia in patients with nr-axSpA (18). In contrast, less marked differences with ASDAS would be expected, since the ASDAS score includes the patient global assessment of disease activity instead of fatigue.

Patients with axSpA are subject to functional impairment, negatively impacting their quality of life (2) and HS. Our study measured HS using the ASAS-HI questionnaire and observed that the impact on HS was low to moderate overall (20), but females had a higher impact on HS than males. While gender differences in HS had been seldomly explored, our results align with two recent studies reporting worse HS in females with axSpA and AS, examined using the same scale (21, 22). Interestingly, disease activity measures were independent determinants for ASAS-HI scores in both genders (22), suggesting that a better disease control (less active disease) may improve HS of AS patients regardless of gender.

Our study has some strengths and limitations. Among the strengths, our results are consistent with those reported in previous studies and are representative of the patients with AS attended in Spanish outpatient rheumatology clinics. However, MIDAS study entails limitations due to its design. The retrospective, cross-sectional design of the study allows a description of the patients' current disease control and health. However, it does not detect changes over time depending on the evolution of the disease. In the present study, the inclusion criteria required stable treatment for the 3 months prior to inclusion. Also, as the study did not require a minimum number of patients per treatment, our results may add evidence regarding the most used treatments at present, but not enough information for newer available therapies for patients with AS. Indeed, it reflects the current clinical practice.

## 5. Conclusion

In this ancillary analysis of the MIDAS study, a higher proportion of females than males with AS have active disease when it is analyzed using the BASDAI definition. However, regarding the use of the ASDAS-CRP score to evaluate disease control, these gender differences are less pronounced. These findings underscore the importance of gender differences when assessing disease activity among patients with AS, which should be kept in mind to improve patients' evaluations and outcomes. Additionally, the impact of disease activity on HS (measured using the ASAS-HI) was higher in females than males with AS.

## Data availability statement

The original contributions presented in the study are included in the article/Supplementary material, further inquiries can be directed to the corresponding author.

## Ethics statement

The studies involving human participants were reviewed and approved by Ethical and Clinical Research Committee of the 12 de Octubre Hospital. The patients/participants provided their written informed consent to participate in this study.

## Author contributions

All authors meet criteria for authorship as recommended by the International Committee of Medical Journal Editors (ICMJE). All authors were involved in drafting the article or revising it critically for important intellectual content, and approved the final version to be submitted for publication.
