# Supplementary material for: Disease activity indexes might not capture the same disease aspects in males and females with ankylosing spondylitis: A real-world nationwide analysis
Source: Front Med (Lausanne). 2022 Dec 21;9:1078325. doi: 10.3389/fmed.2022.1078325 (PMC9811117; doi:10.3389/fmed.2022.1078325)
Supplement: Supplementary file 1 [file Table_1.DOCX]

Supplementary Material

# Supplementary Table

**Table.** Principal Investigators who participated in the MIDAS study

| **Principal Investigators** | **Centre** |
| --- | --- |
| Dr. Mª Luz García Vivar | Hospital de Basurto |
| Dr. Jesús Babío Herraiz | Hospital Universitario de Cabueñes |
| Dr. Xavier Juanola | Hospital Universitari de Bellvitge |
| Dr. Jordi Gratacós | Corporació Sanitària Parc Taulí |
| Dr. Eugenio de Miguel | Hospital Universitario La Paz |
| Dr. Joaquin Belzunegui | Hospital Universitario de Donostia |
| Dr. Anna Pàmies Corts | Hospital Joan XXIII Tarragona |
| Dr. Jose J. Perez Venegas | Hospital Universitario Virgen de Macarena |
| Dr. José Luis Pablos Álvarez | Hospital Universitario 12 de Octubre |
| Dr. Emma Beltrán Catalán | Hospital del Mar |
| Dr. Jesús Sanz Sanz | Hospital Universitario Puerta de Hierro |
| Dr. Andrés Ponce | Hospital General de Granollers |
| Dr. Mª Carmen Castro Villegas | Hospital Reina Sofía de Córdoba |
| Dr. Alejandro Muñoz | Hospital Virgen del Rocío |
| Dr. Ana Urruticoetxea | Hospital Can Misses |
| Dr. José Manuel Rodríguez Heredia | Hospital Universitario De Getafe |
| Dr. Carlos García Porrúa | Hospital Universitario Lucus Augusti |
| Dr. Ignacio Villa Blanco | Hospital de Sierrallana |
| Dr. Carlos J. Rodríguez Lozano | Hospital Universitario Dr. Negrín |
| Dr. Paloma Vela | Hospital General Universitario Dr Balmis de Alicante |
| Dr. Ceferino Barbazán | Hospital Meixoeiro de Vigo |
| Dr. Adela Gallego Flores | Complejo Hospital Universitario de Badajoz |
| Dr. Ruth López González | Hospital Provincial de Zamora |
| Dr. Javier Calvo Català | Hospital General de Valencia |
| Dr. Manel Pujol | Hospital Mutua de Terrassa |
| Dr. Susana Romero | Hospital de Pontevedra |
| Dr. Ana Cruz Valenciano | Hospital Universitario Severo Ochoa |
| Dr. Miguel Ángel Abad | Hospital Virgen del Puerto |
| Dr. Ana Paula Cacheda | Hospital Son Llátzer |
| Dr. Javier del Pino Montes | Hospital Universitario de Salamanca |
| Dr. Fernando Rodriguez | Hospital Santa Lucía de Cartagena |
| Dr. Cristina Fernández Carballido | Hospital Universitario San Juan de Alicante |
| Dr. J. Manuel Moreno | Hospital Universitario Arrixaca |
| Dr. Teresa Clavaguera | Hospital Josep Trueta |
| Dr. Javier González Polo | Hospital Nuestra Sra. Del Prado |
| Dr. Sergio Machín García | Complejo Hospitalario Universitario Insular |
| Dr. Pilar del Río | Hospital Clínico Universitario Lozano Blesa |
| Dr. Patricia Moya | Hospital de la Santa Creu i Sant Pau |
| Dr. Antonio Fernández Nebro | Complejo Hospitalario de Málaga |
